# Complex Networks of Prion-Like Proteins Reveal Cross Talk Between Stress and Memory Pathways in Plants

**DOI:** 10.3389/fpls.2021.707286

**Published:** 2021-07-26

**Authors:** Sampurna Garai, Citu, Sneh L. Singla-Pareek, Sudhir K. Sopory, Charanpreet Kaur, Gitanjali Yadav

**Affiliations:** ^1^Plant Stress Biology, International Centre for Genetic Engineering and Biotechnology (ICGEB), New Delhi, India; ^2^Computational Biology Laboratory, National Institute of Plant Genome Research (NIPGR), New Delhi, India; ^3^Stress Physiology and Molecular Biology Laboratory, School of Life Sciences, Jawaharlal Nehru University, New Delhi, India; ^4^Department of Plant Sciences, University of Cambridge, Cambridge, United Kingdom

**Keywords:** complex network analysis, *Oryza sativa*, prion-like domains, stress biology, stress memory, retrotransposons, transposons, multi-omics

## Abstract

Prions are often considered as molecular memory devices, generating reproducible memory of a conformational change. Prion-like proteins (PrLPs) have been widely demonstrated to be present in plants, but their role in plant stress and memory remains unexplored. In this work, we report the widespread presence of PrLPs in plants through a comprehensive meta-analysis of 39 genomes representing major taxonomic groups. We find diverse functional roles associated with these proteins in various species and term the full complement of PrLPs in a genome as its “*prionome.”* In particular, we found the rice prionome being significantly enriched in transposons/retrotransposons (Ts/RTRs) and identified over 60 rice PrLPs that were differentially regulated in stress and developmental responses. This prompted us to explore whether and to what extent PrLPs may build stress memory. By integrating the available rice interactome, transcriptome, and regulome data sets, we could find links between stress and memory pathways that would not have otherwise been discernible. Regulatory inferences derived from the superimposition of these data sets revealed a complex network and cross talk between PrLPs, transcription factors (TFs), and the genes involved in stress priming. This integrative meta-analysis connects transient and transgenerational memory mechanisms in plants with PrLPs, suggesting that plant memory may rely upon protein-based signals in addition to chromatin-based epigenetic signals. Taken together, our work provides important insights into the anticipated role of prion-like candidates in stress and memory, paving the way for more focused studies for validating the role of the identified PrLPs in memory acclimation.

## Introduction

Plant memory has emerged as one of the most fascinating fields of study in modern science, especially in view of the intricate mechanisms evolved by plants to survive under ever-changing unfavorable and adverse environmental conditions. One such phenomenon is related to the development of “stress memory” in plants, which can occur *via* “priming,” wherein a prior short exposure to stress “primes” the plant for subsequent stress episodes by facilitating a faster and heightened response of resistance (Kinoshita and Seki, 2014; Crisp et al., 2016). Two major mechanisms have been reported to contribute toward priming in plants; one being epigenetic in nature, and this is mediated by nucleosome remodeling *via* chromatin modification and changes in the state of DNA methylation (Brzezinka et al., 2016). In contrast, another mechanism is associated with heritable and self-perpetuating changes in the activity of proteins, mainly prions. The role of prion-like proteins (PrLPs) in plant memory is only beginning to emerge, and these have been associated with diverse stress and memory processes in plants, including flowering time, as well as thermosensory responsiveness (Bailey et al., 2004; Shorter and Lindquist, 2005; Jung et al., 2020).

Prions are a subclass of amyloid proteins, which can act as heritable elements in their aggregated state, constituting self-replicating entities with the ability to perpetuate and transmit over generations. Prions caught the attention of the scientific world through Pruisiner’s pioneering work two decades ago (Prusiner, 1998). These proteins can switch from non-aggregated states to self-templating highly ordered aggregates and transmit the same to other homologous polypeptide sequences. This property allows them to confer stable changes in the biological states that are of great interest in molecular and evolutionary biology (Newby and Lindquist, 2013). Prions have been extensively studied in several organisms (Alberti et al., 2009). Besides being associated with negative effects in yeast (Wickner, 2019) and devastating diseases in humans and mammals (Prusiner, 1998; Imran and Mahmood, 2011; Das and Zou, 2016), they also have a role in conferring adaptive significance (True and Lindquist, 2000; Jarosz et al., 2014; Pham et al., 2014; Chakrabortee et al., 2016a). Prion form of the protein can act as a bet-hedging device to offer a growth advantage under stressful environments, such as Sup35 protein from yeast, the prion form [PSI+] of which is positively selected under stressful conditions in exchange for a reduced overall fitness (Newby and Lindquist, 2013; Franzmann et al., 2018). In humans, diseases such as Alzheimer’s, Parkinson’s, amyotrophic lateral sclerosis, and Huntington’s syndrome are also associated with amyloid proteins, which have certain prion-like properties but unlike typical prion diseases they are not transmissible (Schwab et al., 2008; Nonaka et al., 2013; Iglesias et al., 2019).

A majority of prion proteins are marked by glutamine/asparagine- (Q/N-) enriched prion-forming domains, which are essential as well as sufficient for propagation (Sabate et al., 2015). In addition, these domains also include specific short amyloid-prone sequences, which possibly trigger the conversion of proteins to an amyloid form (Sabate et al., 2015). *In silico* studies, which were carried out using various algorithms such as pWALTZ, prion aggregation prediction algorithm (PAPA), and prion-like amino acid composition (PLAAC), have helped in identifying proteins with these prion-like domains (PrLDs) in different proteomes (Antonets and Nizhnikov, 2017; Iglesias et al., 2019). However, it is to be noted that not all PrLPs harboring these PrLDs may be true prions as they may exhibit only some properties of typical prions.

Plants have the ability to sense cyclic changes in their environment, which may be compared to a memory process wherein PrLPs may provide a unique mode of biochemical memory through self-perpetuating changes in protein conformation and function. As many as 474 PrLPs have been identified in *Arabidopsis Thaliana* proteome (Chakrabortee et al., 2016b), suggesting the need for a more comprehensive identification and general analysis of PrLPs in plants. Interestingly, plant flowering is a significant case for biological memory in Arabidopsis as its regulation involves memorizing and integrating previously encountered environmental conditions. More recently, early flowering 3 (ELF3), a PrLD-harboring protein in Arabidopsis, has been found to confer thermosensory responsiveness, a process that has previously been associated with epigenetic modulation, offering an opportunity for new studies that may bridge the two schools of thought in plant memory mechanisms (Jung et al., 2020).

In view of the above, we have addressed this knowledge gap through the identification and dissection of the possible roles of PrLPs in the plant kingdom (which we collectively term as “prionome”). Collectively, we identified more than 4,479 PrLPs in 39 plants and followed this by a comprehensive genome-wide analysis for PrLP functions in rice. Rice is a model crop, with very well annotated nuclear and organellar genomes, as well as the availability of a large number of high-throughput omics data sets. However, the major reason for our selection of rice for this work was based on the unique features of the rice prionome; high density of PrLPs compared to other species, as well as a significant enrichment in retrotransposon- (RTR-) like genes. Thus, the rice prionome (with 201 PrLPs) was investigated in terms of the available interactome, regulome, and many transcriptomes (such as stress, anatomy, development, and circadian time-series data sets), to understand the possible correlation between the physiological roles of PrLPs and stress priming. For reducing data dimensionality, we then superimposed all three high-throughput omics data sets to generate transcriptional regulatory networks. This comprehensive network analysis revealed distinct clusters among rice PrLPs that have evolved to form intricate linkages with hub nodes represented by transcription factors (TFs), which, in turn, are either positively or negatively regulating genes involved in mediating transient and transgenerational memory in the plant kingdom. We term our findings as a “cross talk” between stress and memory pathways at a genome-wide level in rice. Given the large number of PrLPs that have been identified in other plant species, the patterns we find in rice may prove to be general for the plant kingdom. Taken together, our work is an attempt to unify the well-known epigenetic and lesser-known protein-based plant memory mechanisms and paves the way to further unravel a cross talk between the factors contributing to stress memory in plants.

## Materials and Methods

### Acquisition of Proteome Sequences and Annotation

Proteome sequences of all species used in this study were downloaded from Phytozome^1^ except for *Picea abies* and rice, for which, Congenie database^2^, and Rice Genome Annotation Project^3^ were used, respectively. An annotation for PrLPs above CoreScore ≥ 25 was performed using respective databases.

### Identification of PrLPs

Plant proteomes were analyzed for the presence of PrLPs using the PLAAC software^4^. A minimum length for prion domains (L core) was set at 60 and parameter α was set at 50. For background frequencies, *A. thaliana* proteome was selected. A total number of proteins that contain PrLDs were examined, and the proteins having CoreScore ≥ 25 among these were filtered for further analysis. Density was calculated as the ratio of the identified PrLPs to the total number of proteins present in a particular species. It may be noted here that not all N/Q rich sequences are prions, and our aim was to uncover the most important PrLP candidates in plant proteomes with a greater likelihood of exhibiting prion-like behavior even though prionogenicity does depend to a large extent upon these intrinsically disordered Q/N regions (Sabate et al., 2015). At present, systematic experimental screening for prion-like propagation is lacking for non-N/Q-rich proteins so that current prion search algorithms are largely based on yeast prion domains. The identification of new prion-like candidates in unrelated proteomes will require important knowledge-based program adjustments based on new/future experimental data. Hence, we have used the term putative “PrLPs” in this study. Under these circumstances, we found PLAAC as the best alternative that would allow us to screen for potential PLAAC in the plant kingdom to start with. Furthermore, the PLAAC software has previously been used successfully in plants, for detecting PrLPs in Arabidopsis, followed by experimental confirmation, revealing the presence of yeast-like characteristics in plant PrLPs (Chakrabortee et al., 2016b).

### Functional Enrichment Analysis

Gene ontology (GO) enrichment was performed using Phytozome^5^. For a detailed analysis of *Oryza sativa* PrLPs, “Plant Regulomics^6^” and REVIGO^7^ were used for GO enrichment. Prediction for the subcellular localization of rice PrLPs was carried out using “RiceNETDB^8^.”

### Gene Expression Profiles

Data for tissue-specific, developmental, and stress-based expression profiles of gene-encoding rice PrLPs were analyzed using the GENEVESTIGATOR^®^ platform (GV) as it enables a single-step analysis of transcriptional regulation across thousands of experimental conditions^9^. GV integrates the manually curated and quality-controlled gene expression data from public repositories, apart from integrating proprietary data. GV uses standard normalization methods for scaling between the experiments to make the expression values comparable. We exported the data from the “conditional search tool” of GV, selecting for “data values: ratio of expression potential.” This value is defined as (mean value – signal background)/(expression potential – signal background + epsilon). A small epsilon with a value of 1 is added to the denominator to avoid the division by 0 in cases where the expression potential and signal background are (almost) the same. Expression profiles for 62 of the 201 rice PrLPs were found in GV, and these were used for analysis.

For the gene expression profiles of PrLPs in other plants, we used GEO2R, GEOquery, and LIMMA packages from the R Bioconductor project on the open-source software R v3.6.1 and R studio v1.2.1335 (R Core Team, 2012). GEO2R allows the comparison of two or more groups of samples in a GEO series in order to identify the genes that are differentially expressed across experimental conditions. The results are presented as a table of genes ordered by significance and as a collection of graphic plots to help visualize differentially expressed genes and assess data set quality. For this work, we used plant stress-based experiments with accession numbers GSE18361, GSE43050, GSE6901, E-MEXP-3718, GSE41647, E-MEXP-2401, GSE14275, GSE58603, and GSE19024, in order to extract the expression profiles of rice PrLP-coding genes, as listed in Supplementary Table 1.

### Gene Co-expression Data

A similar overrepresentation of DNA-related functions could be seen in biological processes as well; DNA biosynthesis/metabolism, DNA regulation at transcriptional level and gene expression were the predominant PrLP functions across all phyla (Supplementary Figure 1B). Accordingly, we used the available time-series data [in transcripts per million (TPM) counts] for the rice diurnal range across the day–night cycle comprising 48 samples for 24,957 probe sets (Ferrari et al., 2019). Temporal expression patterns for the members of the rice prionome were extracted from this data set and were used for generating pairwise co-expression data. Expression profiles for only 66 of the 201 rice PrLP genes could be found in this data, and these were extracted using normalized TPM values (rescaled between 0 and 1 *via* min–max scaling/normalization). Combined parametric and non-parametric (Spearman’s and Pearson’s) correlations were calculated for each pair of genes using R package Hmisc (Harrell and Dupont, 2017). Significantly correlated, positive, and negative sets of genes were identified by filtering out all correlations below a threshold cutoff of ± 0.8 and the value of *p* < 0.01. The resulting binary expression matrix was used to generate Corrplots for PrLP cluster identification in R corrplot (Wei and Simko, 2019) by applying average linkage clustering to genes and first principle component distance measurements.

### Generation of Protein–Protein Interaction Network

Protein–protein interactors for the 201 rice PrLPs were identified using the protein–protein interaction (PPI) network generation feature in RiceNETDB (see text footnote 8). The generated network was then visualized using Cytoscape version 3.7.2 (Shannon et al., 2003). All identified interacting proteins were then mapped to the Kyoto Encyclopedia of Genes and Genomes (KEGG) database (Kanehisa et al., 2016) in order to functionally characterize the PrLP interactome.

### Rice Homologs for Stress Memory/Recovery/Acclimatization

The expression profile of genes (including PrLPs) for plant stress and memory data was mined from either the literature studies (Hemme et al., 2014; Brzezinka et al., 2016; Georgii et al., 2019; Zuther et al., 2019; Jung et al., 2020) or the NCBI GEO database for accessions, GSE123072 and GSE112161 using the GEO2R tool^10^, as listed in Supplementary Table 1. For the identification of rice homologs of all non-rice genes reported to be involved in stress or memory responses, local blast against the rice database was performed, followed by filtering of the results by an *e*-value cutoff less than 1e-5, query coverage, and identity cutoffs more than 50 and 35%, respectively, and the selection of the top ranking gene in rice based on the *e*-value. Lists and associated references are provided in Supplementary Table 2. Heatmaps were generated using the R package.

### Construction and Clustering of Gene Regulatory Network

The gene regulatory network (GRN) for PrLPs was generated from co-expression data in a stepwise manner. The first layer of this network included all significant self-correlations among PrLPs. The second layer was formed by adding co-expression data for PrLPs and rice TFs. The third and last layer of the GRN were created by adding stress- and memory-related genes, which are found to be significantly correlated with TFs or RTRs in the rice prionome. Finally, the network was filtered with evidence from *cis-*binding element information, to retain high fidelity regulatory interactions. For this, promoter sequences of 1,700 bp length were extracted from the rice structural annotation (GFF3 format) files for each PrLP. Each such “promoter” comprised 1,500 bp upstream and 200 bp downstream of the annotated transcription start site. TFs that bind to these promoters were identified using the plant transcription regulatory map database^11^ (Tian et al., 2020). This information was used as supporting evidence to filter the pairs of genes that are found to be significantly co-expressed in the binary co-expression matrix of PrLPs. The resulting filtered co-expression matrix was exported to an edge list in a SIF format file and visualized using Cytoscape version 3.7.2 (Shannon et al., 2003). Molecular complex detection (MCODE) clustering was applied on this network to identify high-ranking gene clusters using default parameters (Bader and Hogue, 2003).

## Results

### Chlorophytes Possess the Highest Prion Density in the Functionally Diverse Plant Prionome

**FIGURE 1:**
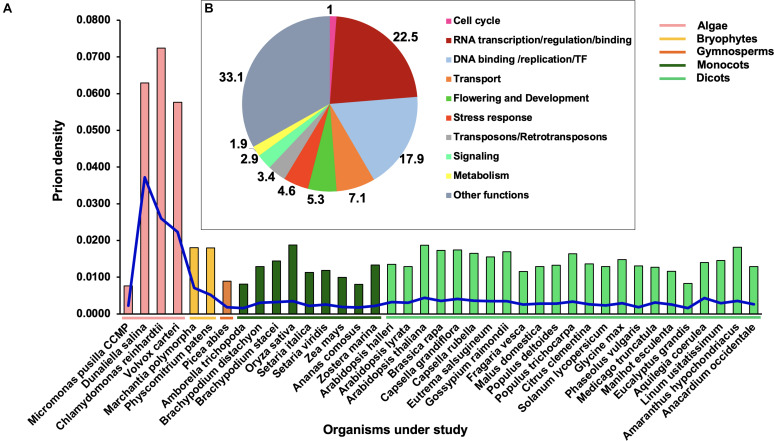
Distribution of prion-like proteins (PrLPs) across the plant kingdom. **(A)** Bars show densities (PrLP count/proteome size), blue lines show a specific prion density at a threshold COREscore value of 25 as obtained through prion-like amino acid composition (PLAAC) software analyses. **(B)** Ten functional categories of PrLPs from 39 plants.

Figure 1A shows the density distribution of PrLPs across 39 plant species. It reveals the wide prevalence of PrLPs across the green lineage, ranging from algae (four chlorophytes) to mosses, ferns (*Marchantia polymorpha* and *Physcomitrium patens*), gymnosperms (Pine), ancient angiosperms (*Amborella trichopoda*), monocots (six grass family members), all the way to dicots (25 in all, representing 13 distinct taxonomic families). A density distribution enables a comparison between the diverse phyla as proteome sizes are hugely variable between species. As can be seen in this figure, algal species possess the highest prion densities (even with stringent prion selection thresholds), with the exception of *Micromonas pusilla CCMP*, which had the lowest (0.0076) density across all phyla. Overall, lower plant taxa possess higher PrLP densities, with *O. sativa* and *A. thaliana* depicting the greatest prion density among all higher plants.

In terms of absolute numbers, we identified 4,479 PrLPs (threshold COREscore value > 25), which were classified into 10 functional categories (Figure 1B). The representation was the highest for the RNA-binding/regulation/transcription (22.5%) category followed by DNA binding/replication/TF (17.9%) and transport-related (7.1%) functions (Supplementary Table 3). Notably, one of the top 10 roles represented by PrLPs included transposons- (Ts/RTRs-) related functions (Figure 1B). These 10 categories were further supported by GO enrichment of plant PrLPs (Supplementary Table 4), with a nucleic acid binding function (GO:0003676) universally enriched across different phyla (Supplementary Figure 1A). Chlorophytes were specifically enriched in various functions related to DNA binding. Further, DNA/RNA-related functions, such as transcription co-regulator activity (GO:0003712) and RNA binding (GO:0003723), were found to be co-enriched in *A. thaliana, P. abies*, and *P. patens*. Flowering and development were overrepresented in various plant prionomes along with the proteins involved in the regulation of the reproductive processes (GO:2000241). Plant prionomes were also enriched in other functions such as aromatic/cyclic compound metabolic process (GO:1901362, GO:0046483, GO:0019438, and GO:0006725 terms) in most species (Supplementary Figure 1A).

In the context of biological processes, similar indications about the over-representation of DNA-related functions could be seen, with DNA biosynthesis/metabolism, DNA regulation at transcriptional level and gene expression, being the predominant biological PrLP functions across all phyla (Supplementary Figure 1B). Nitrogen metabolism-related processes were found to be enriched in higher angiosperms (GO:0051171, GO:0034641, and GO:0044271). An analysis of cellular location revealed the enrichment of various nucleus-related roles of PrLPs (Supplementary Figure 1C). For example, PrLPs form a part of the TFs TFIID complex (GO:0005669) in many prionomes while RNA polymerase II holoenzyme (GO:0016591) and complex (GO:0030880) components could be associated with *Populus deltoides, Malus domestica, Brassica rapa*, and *Senna italica* prionomes.

The unique features of the rice prionome, namely the overwhelming preponderance of Ts/RTRs and the high density of PrLPs compared to other species, led us to explore the rice prionome in more detail, especially from the viewpoint of a possible correlation between the physiological roles of PrLPs and stress priming, as described in the next section.

### The Rice Prionome: Enrichment of Ts/RTRs

**FIGURE 2:**
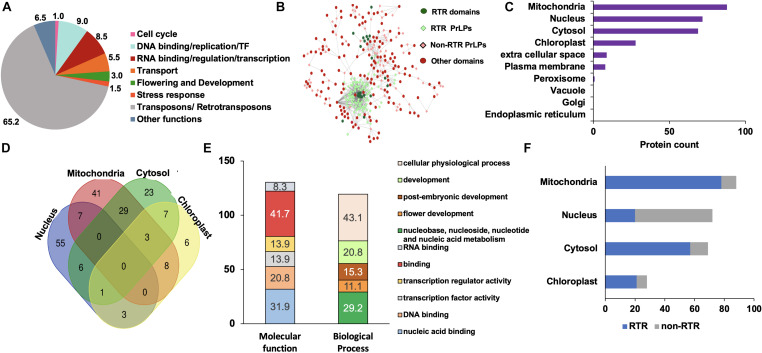
Rice prionome has a preponderance of transposon/retrotransposon (Ts/RTR) type of PrLPs and participates in diverse functions of the cell with organellar enrichment of specific functions. **(A)** Functional classification. **(B)** Visualization of domain categories including transposons/retrotransposons (Ts/RTRs) (dark green circles) in Ts/RTRs PrLP candidates (light green diamonds), other domains (red circles) in non-Ts/RTR PrLPs (light pink diamonds). **(C)** Organelle-based distribution. **(D)** Venn diagram depicting the overlapping distribution of PrLPs in top four organelles. **(E)** Gene ontology- (GO-) based classification of nucleus localized rice PrLPs. **(F)** Percentage distribution of Ts/RTRs and non-Ts/RTRs in different organelles.

Rice remains one of the most extensively studied model crops and serves as an established system with well-annotated nuclear and organellar genomes, supplemented by the availability of a large number of high-throughput omics data sets. We thus used the rice prionome as a representative case study to elucidate the possible roles of PrLPs in plant physiology, and to explore their involvement, if any, in stress or its acclimation or in building stress memory. As listed in Supplementary Table 5, 228 PrLPs (encoded by 201 genes) were identified in the rice proteome. As mentioned earlier, functional classification and GO enrichment of the rice prionome revealed numerous striking features (Figure 2 and Supplementary Figure 2). The most striking feature is the presence of an exceptionally large number of Ts/RTRs in rice prionome (Figures 2A,B and Supplementary Table 5). Notably, more than half of the identified rice PrLPs are Ts/RTRs (65%; 131 of 201). Among other plants, one chlorophyte *Dunaliella salina* (0.3%); three monocots *Brachypodium distachyon* (1.2%), *S. italica* (1.3%), and *Zea mays* (3.9%); and three dicots *A. thaliana* (3.8%), *Gossypium raimondii* (1.3%), and *M. domestica* (3.9%) were found to harbor Ts/RTRs in their prionomes (Supplementary Table 3). We believe these numbers may not truly represent the absence of RTRs in other plants, but rather a lack of sufficient or complete annotation about this important yet under-explored group of proteins, and that future studies may reveal the existence of RTRs in other plant prionomes as well.

In terms of the cellular distribution of rice PrLPs, we observed a preference for mitochondrial localization followed closely by nuclear and cytosol-based localization, with instances of localization in more than one organelle (Figures 2C,D). Nuclear PrLPs were associated with DNA-related roles such as TFs [TCP, auxin-related factor (ARF)], transcriptional activators (RSG and SWIRM domains), flowering regulation-related (LEUNIG and FCA proteins), as well as the RNA-binding FUS proteins and KH domain proteins (Figure 2E and Supplementary Figure 2). Importantly, 12 members in the rice prionome were identified as TFs representing ARF, bZIP, C3H, and NF-YB families. In addition, transport proteins, such as ANTH/ENTH domains, as well as VHS/GAT domain-containing PrLPs were also predicted to be nucleus-localized (Supplementary Table 5). In terms of biological processes, rice PrLPs were found to be enriched in the regulation of flower development (GO:0009909), auxin activation pathways (GO:0009734), and G-protein-coupled receptor signaling (GO:0007186) (Supplementary Figure 2). Molecular function enrichment highlighted ATP-dependent helicases (GO:0008026), lipid binding (GO:0008289), and its involvement in the nutrient reservoirs of cells (GO:0045735) (Supplementary Figure 2). Notably, Ts/RTRs constituted more than 80% of the cytosolic and mitochondrial rice prionome (Figure 2F). For rice, the available annotation (RGAP version 7) has enabled a thorough investigation of the rice prionome, especially in terms of its unique enrichment for RTR/Ts domains.

Annotations and GO enrichment highlighted the potential regulatory and DNA-binding roles of PrLPs. This led us to investigate the conditional and/or differential expression profiles of these 201 genes, which have been addressed in the next section.

### Rice Prionome: Gene Expression Profiles

**FIGURE 3:**
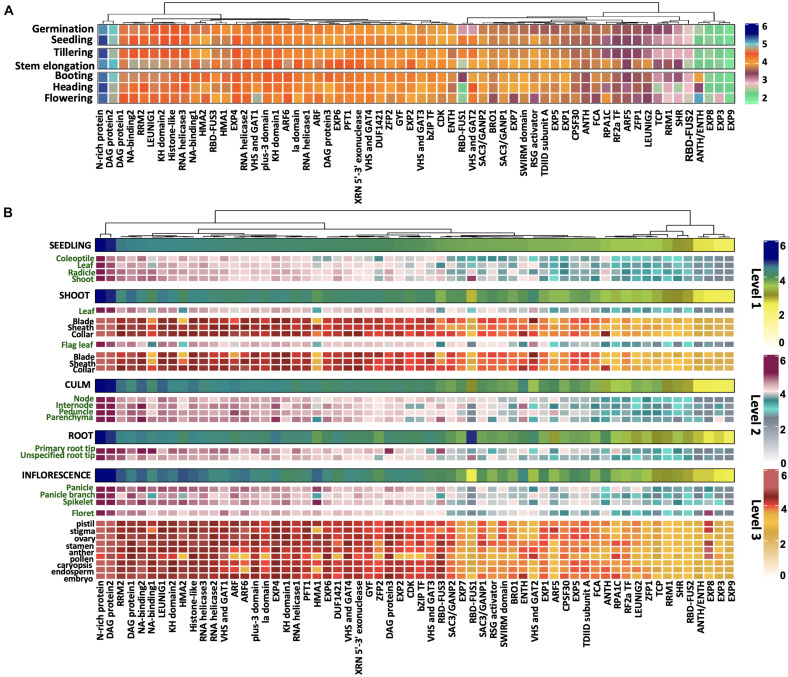
Differential expression of rice prionome across **(A)** developmental stages and **(B)** across specific organs and tissues of rice. Expression profile has been depicted in organs (first level), their parts (second level), and different tissues (third level) within these parts. The profile of each of the three levels has been indicated by a different colored scale. Data are shown for 62 PrLPs obtained from Genevestigator. For full names and accession IDs, refer to Supplementary Table 5.

In order to gain deeper insights into the potential prion-like roles of PrLPs, we analyzed the development and tissue-specific expression profiles of rice PrLPs. As described in methods, expression profiles could be detected for only 62 of the 201 PrLPs in the rice prionome and these are depicted in Figure 3. Comparing across all stages of development, expression levels were the highest for the stress-responsive N-rich protein and DAG protein-2, whereas these values were the lowest for RBD-FUS2, EXP3, EXP8, and EXP9. However, significant variations were noted for RNA-binding RBD-FUS1 protein, which was more abundant at seedling and tillering stages and significantly downregulated at the flowering stage. Further, the transcript levels of ANTH/ENTH and RPA1C protein were increased at the flowering stage while those of BRO1, RSG activator and VHS, and GAT1 were markedly downregulated during flowering. The TCP domain-containing protein, SHR TF, RRM1, ZFP1, and floral homeotic gene LEUNIG2 were upregulated in the stem elongation stage and downregulated at the heading stage. In coherence with the flowering and developmental functions, FCA has differential expression during vegetative and flowering stages (Figure 3A).

We also examined the expression profiles of rice PrLPs across different tissues as shown in Figure 3B. The most highly expressed group of PrLPs comprised of N-rich protein and DAG protein2, whereas SHR, RBD-FUS2, ANTH/ENTH, and EXP3/8/9 constituted the least expressing groups across all tissues, as also observed across developmental stages. Differential expression of PrLPs was observed between male and female reproductive tissues, where ZFP2, ARF5, SWIRM domain, and SAC3/GANP1 showed much lesser expression levels in male tissues (stamen, anther, and pollen) as compared to female tissues (pistil, stigma, and ovary). Also, bZIP TF, CDK, and ARF genes, which were otherwise highly abundant in different organs, showed lower expression in pollen, whereas RPA1C showed the opposite pattern of expression. Interestingly, stamen had upregulated the expression for NA binding1 and RSG activator. The RBD-FUS1, however, showed greater expression in roots than in shoots and inflorescence. In contrast, RRM2, NA-binding1, HMA1, and HMA2 were expressed more in the inflorescence. Further, VHS and GAT2 showed the lowest expression in roots, particularly in the primary root tip, and the highest expression in leaf blades. Importantly, root tips were particularly seen to express RBD-FUS3 and NA-binding proteins.

Overall, we noted that the measurable rice prionome (62/201 PrLPs for which data were available) is extensively expressed in diverse tissues across developmental stages but has a variable expression in the case of the genes known to be involved in regulatory roles, especially for flowering and reproductive pathways. In order to find additional evidence of PrLP involvement in these pathways, and to elucidate other functional pathways in which PrLPs participate, we investigated the PPI space for PrLPs, as well as their expression profiles under different kinds of stress, as described in the next two sections.

### Rice Prionome Interaction Network

**FIGURE 4:**
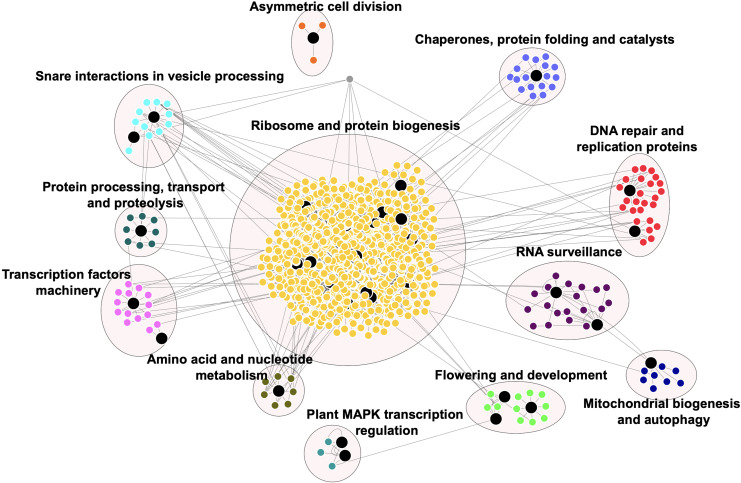
Rice prionome interaction network indicates the role of PrLPs (large black nodes) in essential cellular processes. Different clusters depict the proteins involved in the specific processes. Protein–protein interaction (PPI) data were obtained from RiceNETDB and a network was generated through multiple correspondence analysis (MCA) clustering in Cytoscape.

Figure 4 shows the PPI network for the available rice prionome. As in the case of transcriptome data, physical interacting partners have been reported for only 37 of the 201 PrLPs to date, revealing the need for more data in the public domain for model species. In all, we detected 1,263 binary interacting partners for PrLPs, which were then mapped on to the KEGG database, revealing the observations that are consistent with the previous section, showing a preponderance of DNA- and RNA-binding processes, as well as functional clusters involved in ribosome and protein biogenesis, transcriptional machinery and its regulation, DNA replication/repair, and RNA surveillance (Figure 4). The largest cluster was related to ribosome and protein biogenesis, represented by 886 proteins including 16 rice PrLPs. Similarly, flowering and development were also represented as a significant functional category in the interactome, along with subclusters representing mitochondrial biogenesis and autophagy, plant mitogen-activated protein kinase (MAPK) signaling pathway and nucleotide, and amino acid metabolism-related functions. Thus, evidence from PPI maps reinforced the regulatory roles of PrLPs, and this led us to investigate the response of the rice prionome to biotic and biotic stresses as described in the next section.

### Role of PrLPs in Stress and Memory

**FIGURE 5:**
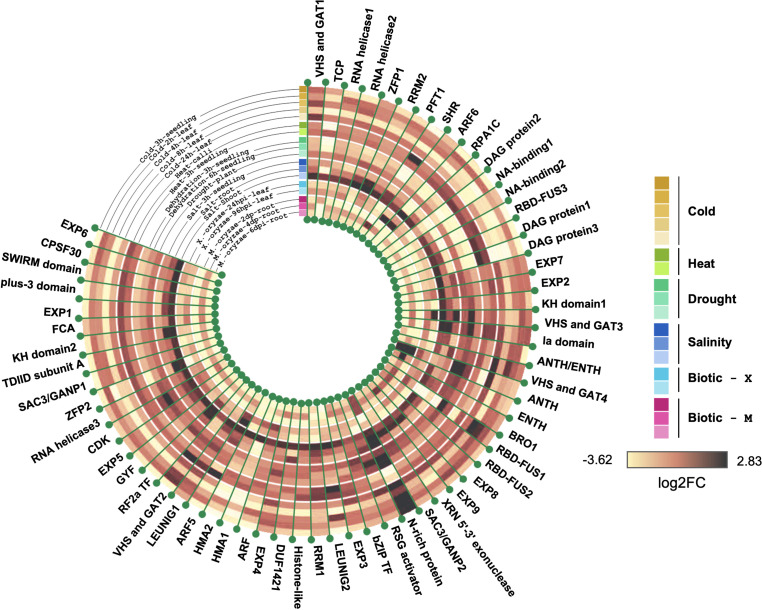
Rice prionome possesses members responsive to stress conditions (eight distinct abiotic and biotic treatments). Tree constructed using R shows log2 fold change in expression. Data have been obtained from the Genevestigator platform. Different shades of a particular color have been used to represent different conditions imposed for giving that particular stress.

Figure 5 shows the stress-responsive profiles of 62 members of the rice prionome for which the expression data were available. An assessment of the rice PrLP stress transcriptome identified specific PrLPs whose expression was significantly altered in response to stress conditions. Previous reports of involvement of PrLPs in stress memory have mainly focused on thermostress, and we also found evidence for this in our data. For example, heat stress resulted in a nine-fold decrease in transcript levels of N-rich protein, whereas cold stress led to the downregulation of RBD-FUS3, NA-binding1, DAG protein1, HMA1/2, and ZFP2 concomitant with the upregulation of RBD-FUS1, and N-rich protein (earlier noted to be the most stress-responsive PrLP). Similarly, PrLPs encoding transcriptional corepressor LEUNIG1, auxin response factor ARF5, DAG protein1, VHS and GAT1, and SHR protein showed increased in response to heat treatment while the expression of TCP and ANTH genes was more than four-fold downregulated under high temperature.

We also found other stresses like drought, salinity, and *M. oryzae* infection resulting in a marked upregulation of the N-rich protein. In addition, the ANTH transcript levels are also drought-inducible, while only two PrLPs (N-rich protein and VHS and GAT protein1) appear to be salt-inducible. Biotic stress responsiveness of the rice prionome was only observed for N-rich protein and ANTH. Overall, stress profiling of the rice prionome suggested differential regulation catering to diverse processes from development to stress.

Interestingly, we found additional evidence for the role of PrLPs in stress pathways, by investigating plants other than rice. This was made possible by the availability of transcriptome data sets related to abiotic stress memory and stress recovery in several other plants. We checked the expression profiles of some of the abovementioned rice PrLP homologs, as depicted in Supplementary Figures 3A–E. The data includes cold stress in Arabidopsis (Zuther et al., 2019), hormonal stress priming in *M. domestica* (Supplementary Figure 3B), etc. PrLP expression profiles in memory responses pertaining to the recovery phase have been observed in *Populus* spp., in response to periodic and successively increasing drought or chronic phase of combined drought-heat stress followed by 1 week of recovery phase (Supplementary Figure 3C). Likewise, heat stress showed a memory response among PrLPs in *Chlamydomonas* and *Arabidopsis* as well (Supplementary Figures 3C,D). Interestingly, we could detect homologs for ten of these genes within the rice prionome supporting the likely role of rice PrLPs in memory signals. These observations when combined with a large number of rice PrLPs impacted by heat stress as mentioned above suggests an important role of the prionome in heat stress and memory. Notably, the very recent reports of heat shock proteins being important epigenetic mediators of transient as well as transgenerational memory led us to explore cross talk between the reported epigenetic signals of memory and signals mediated by PrLPs, as described in the next section. Genes reported to be involved in plant stress or memory are listed in Supplementary Table 2.

### Transcriptional Regulatory Inferences From Gene Co-expression Data

The analysis of individual gene expression profiles cannot reflect cross talk between pairs or groups of genes, but it is possible to derive this information from the extent of correlation between the expression profiles of two or more genes. Furthermore, the reliability of co-expression patterns depends greatly on the number of transcriptomic samples being compared, making it unfeasible to use the conditional expression profiles analyzed in the previous sections. In order to identify statistically significant co-expression values among and between PrLPs and other rice genes, we used the rice diurnal gene expression data set containing 48 samples, as described in section “Materials and Methods.” We could only find temporal expression profiles (listed in Supplementary Table 6) for 66 of the 201 PrLPs. Importantly, these 66 genes included 11 of the 12 TFs present in the rice prionome as well as 5 Ts/RTRs, thereby enabling a thorough investigation of the regulatory role of both TFs and RTRs in the prionome. The genes found to be significantly correlated with PrLPs were used to (a) identify correlated clusters of genes if any, among PrLPs, (b) capture the intersection between PrLPs (especially Ts/RTRs), and the genes known to be involved in stress or memory, and (c) identify TFs defining or influencing the rice prionome for insights into the PrLP master regulatory network.

**FIGURE 6:**
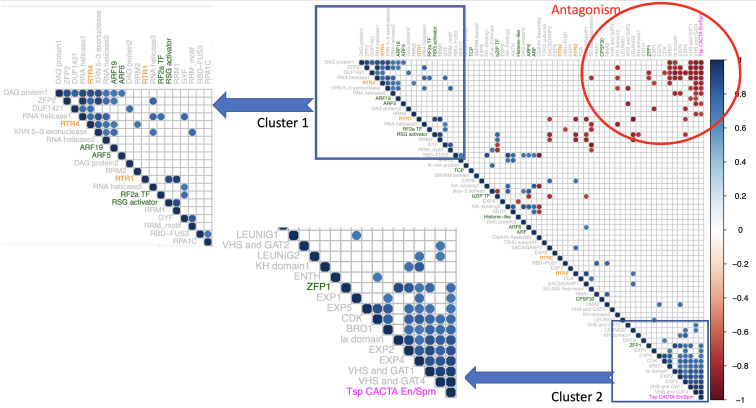
Correlogram of 66 rice PrLPs (listed in Supplementary Table 6). Only significant correlations are shown (cutoff + – 0.8 at *p* < 0.01). Rows/columns ordered by first principal component. The color of PrLP names depicts transcription factors (TFs; green), retrotransposons (RTRs; orange), and Tsp (magenta). Insets show two distinct gene clusters, both with strong intra-cluster synchrony, but both clusters are mutually antagonistic, as can be seen in the upper right corner of the Correlogram (region in red circle).

Significant correlations were observed within the rice prionome (Figure 6) at a correlation coefficient cutoff of ± 0.8 and *p* < 0.01. As can be seen in the figure, two clusters of PrLPs are discernible with about 15 genes in each cluster. All 91 interactions are listed in Supplementary Table 7. Notably, both clusters are significantly negatively correlated with each other suggesting an antagonism in their roles/involvement, with one cluster including four TFs and two Ts/RTRs while the other cluster having one TF and one Ts/RTR. Interestingly, the first cluster has several RNA helicases and exonucleases as well as PrLPs noted to be among the most highly expressed (DAG proteins) and those upregulated in floral tissue, both male (RSG) and female (ARF5 and ZFP2). In contrast, the second cluster showing a negative correlation with the first one contains the BRO1 and RSG activators, whose expression decreases during the flowering stage (Figure 3A). Notably, this cluster also has the two LEUNIG proteins that repress several floral homeotic genes in the floral meristems, required for proper differentiation of stamen and carpel structures in the flower (Franks et al., 2002; Sridhar et al., 2004). These patterns suggest a role for PrLP-mediated regulation among flowering genes. As can be seen in the inset, the first cluster also has several genes that were observed to be downregulated in cold stress (RBD-FUS3, DAG, and ZFP2) and upregulated during heat stress (ARF5 and DAG1).

The composition of the above two PrLP clusters in diurnal co-expression data and the pattern of distribution of their respective TFs, corroborated by observations from condition-specific, tissue-based, and developmental gene expression profiles motivated us to derive the regulatory inferences from co-expression data for all TFs in the rice prionome. About 11 of the 12 TFs in the rice prionome, which had diurnal expression profiles, showed a significant positive correlation with 100 other TFs in rice over the entire day–night cycle as well as a significant inverse correlation (core value < − 0.8 at *p* < 0.01) with another 101 TFs, suggesting a master regulatory role for these 11 members of the rice prionome. To further ascertain a master regulatory role, we checked the upstream regions of all the positively and negatively correlated TFs for the presence of *cis-*binding elements for the eleven PrLPs. This resulted in the identification of 40 high-fidelity rice TFs that had a strong positive or negative correlation with the rice prionome, in addition to containing the respective TF binding sites on their promoter sequences. These TFs have been added to Supplementary Table 7. These high-fidelity TFs belong to the WRKY, Dof (DNA-binding one finger), C2H2 (Cys2/His2-type), Myb-related, lateral organ boundaries domain (LBD), TCP, GATA, G2-like, TriHelix, SQUAMOSA promoter binding protein (SBP), related to ABI3 and VP1 (RAV), and no apical meristem (NAM), *Arabidopsis* transcription activation factor (ATAF), cup-shaped cotyledon (CUC) (NAC) families, while 8 of the 40 are PrLPs themselves, further supporting internal cross talk and diverse regulatory roles of the rice prionome.

### Master Regulatory Network Reveals PrLP Clusters in Memory Acclimation

In order to visualize the patterns of interaction and regulation within and between the rice prionome and sets of other rice TFs or stress or memory genes (for which we had diurnal expression profiles), we constructed a transcriptional regulatory network starting from gene co-expression data, in a stepwise manner, as described in methods. This network enabled us to explore cross talk between TFs and RTRs in the prionome, and the extent to which they may regulate or be controlled by other activators or repressors, especially in stress or memory acclimation.

**FIGURE 7:**
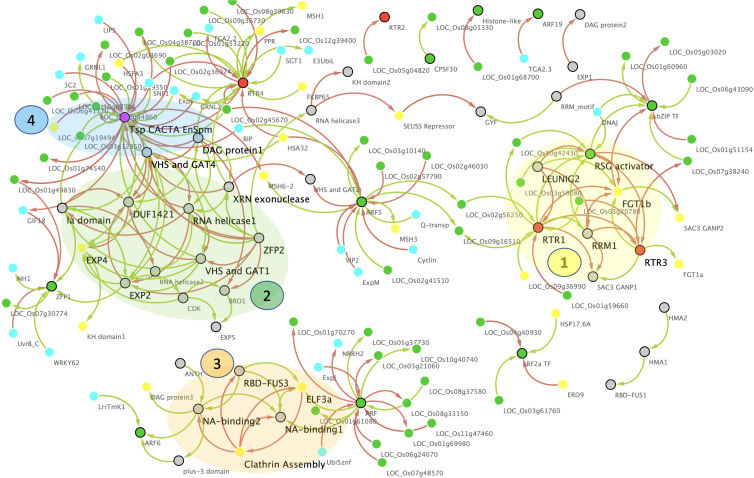
Master gene regulatory network (GRN) of the rice prionome. Nodes represent PrLP genes (black border) and their correlated partners (no border) while edges represent significantly positive (green) or negative (red) expression correlations. Colors of nodes represent transcription factors (TF; green), RTR (orange), and transposon CACTA (magenta). Note the strong overlap of GRN with memory (yellow) and stress (cyan). Four molecular complex detection (MCODE) clusters highlighted in transparent shades, regulatory Hubs in large font.

The five Ts/RTRs in the rice prionome were found to be positively co-expressed with 91 TFs and negatively co-expressed with 77 distinct TFs. Of these pairs of co-expressing TF partners, we performed the same filtering as was done for 11 TFs above, to identify/retain only high-fidelity TFs that have a known binding site on the respective Ts/RTR promoters (Supplementary Table 7). This was achieved by scanning the upstream regions of all co-expressing partners for the presence of *cis-*elements, leading to the retention of 22 true positive TFs. These TFs were added to the rice prionome co-expression data to generate a regulatory network. In the next step, the network was expanded by adding the 32 additional TFs that were identified earlier as high-fidelity co-expressing partners of 11 TFs in the prionome. This network was then superimposed with available information on genes involved in stress or memory acclimation by adding co-expressing partners of Ts/RTRs and TFs in the rice prionome that were (a) present in the rice stress interactome (Wierbowski et al., 2020) or (b) implicated in memory acclimation (Supplementary Table 2). The resulting master regulatory network is depicted in Figure 7, and the corresponding annotated edge list is provided as Supplementary Table 8.

As can be seen in Figure 7, the network has 208 edges and 139 nodes depicting all 66 PrLPs, including 5 Ts/RTRs and 11 TFs, along with other significantly correlated TFs as well as rice genes directly or indirectly implicated in memory and stress events. Most importantly, this network has two large disconnected components, each highlighting the role of rice prionome members as hubs for the currently known data on memory acclimation. Four top-ranking MCODE clusters have been highlighted on the network and each is composed of distinct but tightly interconnected PrLP genes. Interestingly, only two non-PrLP genes are hubs in these four clusters. These are homologs of Arabidopsis ELF3 and FORGETTER1 (FGT1) genes that have very recently been associated with heat stress memory *via* PrLDs (Jung et al., 2020) and chromatin remodeling mechanism (Brzezinka et al., 2016), respectively. Furthermore, to date, there has been neither been any report connecting these two genes nor the two abovementioned memory mechanisms, while the GRN in Figure 7 clearly depicts how pervasively PrLPs act as bridges between various clusters. For instance, FGT1 lies in the first PrLP cluster where it is closely interacting with RTR1, LEUNIG2, RTR3, RSG activator, and RRM1 in the rice prionome while ELF3 forms a part of the third cluster in the GRN (Figure 7). It is interacting with PrLPs involved in clathrin assembly, NA-, and RNA-binding (RBD-FUS), indicating a possible cross talk between the prion-mediated and epigenetic memory pathways. Other prominent hubs in the PrLP regulatory network are the TFs ARF5, and RF2a as well as Ts/RTRs (CACTA and RTR4), earlier observed in the PrLP correlation plot (Figure 6). These TFs are upregulated in heat stress and female flowers. The four clusters form distinct yet synergistic sub-networks of stress memory within the master regulatory GRN of the prionome, with transposon CACTA, ARF, and RBD-FUS3 having a predominantly antagonistic effect on most memory-related genes. For example, TF ARF5 is positively correlated with two rice homologs of the MutS homolog (MSH) family that has very recently been implicated in transgenerational heat shock memory (Wierbowski et al., 2020) while it also negatively regulates a homolog of the heat shock protein, heat shock associated (HSA32) (Charng et al., 2006; Baurle, 2016). The Ts/RTR family member RTR1 is strongly correlated with a rice homolog of a *Chlamydomonas* gene shown to be involved in stress recovery (Hemme et al., 2014). On the other hand, RTR4 is positively correlated with the rice homolog of MSH1 while being inversely correlated with HSA32 thus, closely mimicking ARF5. Similarly, RF2a expression is strongly positively correlated with multiple rice homologs of Hsp17 and inversely correlated with the rice ERD9 gene, reported to be involved in heat stress memory in Arabidopsis (Izadi et al., 2017). In stark contrast, the transposon CACTA is negatively correlated with HSFA3 and MSH family genes and several heat shock promoter elements while being positively correlated with HSA32. Overall, the GRN of PrLPs reveals a strongly interconnected pattern of interaction between TFs, RTRs, and the genes involved in stress and memory processes, apart from identifying clusters and hubs for future investigation of cross talk between these molecular factors.

## Discussion

This study began with the premise established by evidence emerging from recent experimental reports in plants where PrLPs were shown to have a role in plant memory through their involvement in the regulation of flowering time, as well as thermosensory responsiveness (Bailey et al., 2004; Shorter and Lindquist, 2005; Jung et al., 2020). If these findings prove to be general across the plant kingdom, the outcome would have profound implications in the field of plant memory and cognition, given the high potential density of PrLPs in plants. This was further supported by the high numbers of PrLPs already reported in the *A. thaliana* proteome (Chakrabortee et al., 2016b). The availability of an increasingly large number of plant genome sequences at various stages of completion further strengthened the feasibility of conducting a comprehensive identification and general analysis of PrLPs in plants. The past two decades have witnessed several milestones in experimental and predictive prion research in the animal kingdom, but knowledge about such proteins and their role in plants remains limited (Villar-Piqué et al., 2010). For example, prions are a subclass of amyloid proteins that can give rise to at least two distinct, stable conformational states of which, a self-sustaining amyloidogenic form is dominant. A few studies on prion-(like) proteins suggest that the non-prion (or non-aggregated) form of the protein is a part of a normal cell, serving important physiological functions, such as memory processes, transcriptional and translational machinery, nutrient acquisition, and stress responses (Shorter and Lindquist, 2005). However, the conversion to prion form modifies their function in a way, which can be either harmful or beneficial for the organism. Harmful effects usually result in protein inactivation or the gain of toxic function, leading to various neurological disorders (Prusiner, 1982, 1998, 2012) or an increase in pathogenicity/lethality due to the derailment of homeostatic physiological functions. In contrast, the beneficial prion-like state is certainly not toxic and need not always cause protein inactivation. Beneficial aspects include conferring a survival advantage to the organism under stress apart from the stabilization of memory (Si et al., 2010; Newby and Lindquist, 2013; Si, 2015). The Orb2A gene (a CPEB ortholog) in *Drosophila* possesses prion-like behavior and is involved in the persistence of memories related to mating (Majumdar et al., 2012).

Accordingly, we performed a genome-wide identification and analysis of PrLPs or “prionome” in 39 plants. These 39 species covered a wide range of taxa from mosses to trees, enabling us to derive general patterns about functional divergence based on gene ontologies (Figure 1). The wider phylogenetic distribution of PrLPs, combined with the fact that the algal members of the green lineage had the highest densities, suggested that self-templating conformational states have been conserved all the way from chlorophytes to angiosperms, possibly offering an evolutionary advantage during the evolution of plants. With such high densities, amyloidogenic PrLPs in moss genomes may have contributed to biofilm formation as the determinants of high mechanical resistance of exopolysaccharides (Mostaert et al., 2009), also seen in bacteria (Romero et al., 2010).

This was followed by a detailed GO enrichment analysis of all plant prionomes that offered insights into functional diversity, we found chlorophyte PrLPs to be enriched in signaling-related genes especially, MAPK, protein kinases, and response regulators. Overall, we observed an over-representation of nucleic acid-binding functions among the amyloid-forming proteins, with 40% of the plant prionome having RNA-/DNA-binding functions or nuclear localization. This appears to be a common characteristic of PrLPs, having been previously reported in other organisms (Silva and Cordeiro, 2016; Iglesias et al., 2019). Another category of proteins in the plant prionomes (that has never been reported in previously predicted prionomes) was that of Ts/RTRs, constituting a 65% share in rice alone (Figure 2). Interestingly, indications about prion-like roles of Ts/RTRs do exist in human and animal brain studies, where transposon activity has roles in pathogenesis (Mustafin and Khusnutdinova, 2018). We also found plant prionomes to be enriched in developmental processes like flowering (e.g., SEUSS, PFT, FCA, and LEUNIG proteins), and this is reminiscent of experimental reports where Arabidopsis Luminidependens (LD) that was shown to exhibit prion-like behavior and to mediate plant memory during flowering (Chakrabortee et al., 2016b).

Because the prion-like behavior of proteins has been reported to help in the persistence of “long-term memory” in organisms (Kandel, 2012), we hypothesized that plant PrLPs may also possess a potential role in stress and memory consolidation, and this aspect is discussed here. Stress memory can be short-term (within a generation) or transgenerational (Slaughter et al., 2012; Kinoshita and Seki, 2014; Wibowo et al., 2016). We found several RNA metabolism-related proteins in plant prionomes and it may be noted that in the past decade, RNA-binding proteins viz. RBDs, KH domain-containing proteins or RNA recognition motifs (RRMs) are slowly emerging as the key regulators of plant responses to environmental constraints (Chinnusamy et al., 2007; Ambrosone et al., 2012; Owttrim, 2013). For instance, RRMs function as chaperones in heat stress (Kang et al., 2013), whereas CPSF30 is implicated in redox signaling (Van Ruyskensvelde et al., 2018), and KH domain-containing protein is an important upstream regulator for thermotolerance (Guan et al., 2013).

The preponderance of DNA-binding proteins in the plant prionomes added a new dimension to the anticipated roles of PrLPs in the consolidation of plant stress memory. For example, SWIRM domain-containing proteins have been shown to play a role in temperature-induced shaping of epigenetic memory in Norway spruce (Yakovlev et al., 2016). Further, the intracellular transport-related SAC3/GANP group of proteins are found to be upregulated during the recovery stage of low-temperature stress imposed in *P. vulgaris* seedlings (Badowiec and Weidner, 2014). In *Medicago*, the SAC3/GANP family is, in fact, associated with ABA upregulation within the co-expression sub-network of seeds from the plants subjected to salinity stress, thereby providing evidence for transgenerational plasticity (Vu et al., 2015).

Another mechanism of memory formation in plants is *via* alterations in chromatin states such as DNA methylation, histone tail modifications, or paused RNA polymerase II, which can further modify the patterns of gene expression that underpin memory responses (Avramova, 2015). In this regard, we found several plant PrLPs to be involved in chromatin remodeling (e.g., RNA polymerase II subunits, bZIP, histone methyltransferases, etc.). In fact, RNA polymerase II emerged as an enriched cellular component in the prionomes of several plant species. Further, we found members of the MSH1 gene family, a potential epigenetic sensor of stress (Yang et al., 2020) to be strongly correlated with TF-type PrLPs, suggesting that PrLP-based TFs may act as master regulators of plant memory.

The overwhelming majority of Ts/RTRs in rice prionome led us to investigate the rice prionome in detail, especially from the viewpoint of implications in stress memory. Long terminal repeat (LTR) of plants has previously been reported to be activated under various stress stimuli despite being devoid of specific stress-responsive sequences (Alzohairy et al., 2014; Grandbastien, 2015). Further, the transposition of a heat-activated retrotransposon “ONSEN” has been shown to confer transgenerational ABA insensitivity and subsequently stress tolerance (Ito et al., 2016). Apart from the preponderance of TS/RTRs, the rice prionome was most suitable as a case study for us, due to the presence of several noteworthy molecular factors among rice PrLPs that may be involved in the stress memory and acclimation process. For instance, the regulation of RTRs has been shown to be mediated by the TCP family of TFs playing inhibitory roles in cell division or as positive regulators of aging (Zheng et al., 2018), a phenomenon well known in humans to be influenced by prionization. We found the homologs of TCP family TFs in the rice prionome, apart from flowering regulators such as ARF6 and bZIP. The rice prionome also has LEUNIG1 and SUESS repressor, along with several homologs of memory-associated proteins reported in other plants such as KH domain1, SAC3/GANP, DAG protein, and FCA. Most of these TFs have been reported to be involved in heat stress acclimation but considering the limited information available, it is possible that in the future, more PrLPs associated with biotic as well as abiotic stress memory are discovered. Interestingly, just like proteins, metabolites such as polyamines may also contribute toward the restoration and enhancement of metabolic memory in plants (Mattoo and Handa, 2008; Minocha et al., 2014).

An analysis of the rice prionome (201 PrLPs) revealed diverse biological processes, with variable localization patterns (Figure 2). In fact, several PrLPs were predicted to exhibit potential dual or even tri-compartment localization, of which, RTRs comprised the major fraction, many predicted to be mitochondrial, chloroplastic, or cytosolic. Such organelle-localized PrLPs may facilitate a feedback mechanism by transmitting signals from the organelle to the nucleus under stress conditions, in turn modulating gene expression for the survival of the organism. TLS/FUS, an RNA-binding PrLP from humans, also shows dual localization engaging in nucleo-cytoplasmic shuttling, being primarily localized in the nucleus, but under oxidative stress forms cytoplasmic stress granules (Yamaguchi and Kitajo, 2012).

We then looked for additional evidence for a potential role of PrLPs in stress memory by an analysis of individual PrLP gene expression profiles in tissue-specific, developmental, and stress data sets, as well as in PPI data sets (Figures 3–5). In all of these studies, we found repeated instances of PrLP differential expression and regulation during stress, even though data were limited (only 66 PrLPs had any expression profiles, while only 37 had PPI interactors reported). Interestingly, we were also able to find evidence for differential PrLP expression in memory-related transcriptome data sets of other plants, not just rice (Supplementary Figure 3). This led us to search for co-expression patterns as individual expression profiles cannot provide an indication of pairwise or groupwise patterns between the genes. However, co-expression strengths cannot be extrapolated reliably from limited condition-specific data sets like the ones we used for studying individual expression profiles, and therefore we looked for expression data sets with maximum possible samples in one experiment. The high-resolution rice circadian transcriptome (a 24-h diurnal gene expression data set with 48 samples in 1 experiment) was used for measuring the extent of correlation between pairs of PrLP genes. As observed earlier, expression profiles could only be detected for 66 of the 201 PrLPs in this data set, but this analysis further supported the patterns we had found earlier with individual expression profiles. Among the genes significantly correlated with PrLPs, we found those previously reported to have been involved in stress regulation and stress memory. In addition, co-expression analysis allowed us to identify two antagonistic gene clusters among PrLPs (Figure 6).

Finally, we have attempted to converge the two current schools of plant memory acclimation mechanisms namely, chromatin-based signals and prion/protein-based signals by conducting a detailed GRN analysis of PrLPs that are TFs and RTRs. The availability of high-resolution circadian transcriptome of rice combined with the available data on TF binding *cis-*elements enabled a superimposition of the previously reported plant stress and memory data sets. We generated a high-fidelity GRN for the rice prionome using available gene expression profiles, filtered by the regulome (promoter binding site information) and interactome (reported physical interactions). Among these interactions, we deciphered an intricate master regulatory network of prionome TFs and Ts/RTRs, with at least 50 other rice TFs as well as the reported stress and memory pathways. Most importantly, this work revealed four interconnected clusters comprising the genes known to be involved in both epigenetic- and prion-based signals, involving heat shock memory as well as flowering memory acclimation. The current regulatory network (Figure 7) also has several smaller disconnected clusters of PrLPs but this may be due to the limited availability of rice prionome gene expression profiles.

Taken together, we hypothesize an important role for PrLPs in mediating stress memory based on a combination of clues collated from (a) functional annotation and localization, (b) GO enrichment, (c) stress memory specific expression profiles in rice and other plants, (d) interactome (PPI) data, (e) promoter binding (regulome) data indicating the presence of known upstream *cis-*elements, (f) unweighted co-expression network analysis, and finally, and (g) GRNs generated by the superimposition of all data sets. It must be noted that knowledge-based approaches are dependent on the available knowledge by definition, and despite choosing one of the most widely used model plant species with a considerable amount of omics data sets, the networks reported in this work could only be generated with the subset of PrLPs for which corresponding data were available (approximately one- third of the rice prionome). However, it is remarkable that even with just one-third of PrLPs, we could identify the patterns and relationships within and between PrLPs (TFs and TS/RTRs) and the genes known to be involved in plant memory pathways. All regulatory inferences have been extrapolated from the available gene expression profiles of ∼30% PrLPs, and yet these domains appear to serve as bridges between epigenetic- and prion-mediated pathways. We believe this to be a strong motivation for the scientific community to conduct more high-throughput studies in model plants. We hope that more detailed rice stress transcriptomes in the future would fill the gaps and fully connect this network, paving the way for PrLPs as important mediators that converge the two currently known memory mechanisms.

## Concluding Remarks

The extraction of biologically meaningful patterns using data integration is now emerging as one of the most evolving fields in modern data science but there are very few such studies for plants (Ding et al., 2020; Zhang et al., 2020). This work began with an aim to identify and investigate the plant prionome in order to explore a general role in memory based on the previous but limited reports of PrLPs having a role in flowering and plant memory. To our surprise, we detected a widespread occurrence of PrLPs across all plant taxa that too after incorporating very stringent thresholds in our search parameters to minimize false positives. Plant PrLPs appear as proteins with diverse functions and a widespread abundance from the lowest plant forms all the way to flowering angiosperms. Interestingly, we also found a significant enrichment of Ts/RTRs (>60%) in the rice prionome leading us to wonder whether the epigenetic- and protein-based memory signals in plants converge through the RTRs. As reported here, we found that rice PrLPs are not only upregulated during various kinds of stresses but also that certain PrLPs (having TS/RTRs or TFs functions) act as “hubs” or key nodes in the rice co-expression networks, thereby acting as the mediators of stress and memory connections, which we term as “cross talk” between the two pathways. Overall, our work dissects a possible link between stress and memory in plants, which may be executed by the mediation of prion-like candidates specifically Ts/RTRs and TFs, thereby helping plants to fortify defenses for a stronger or more rapid response in the future by retaining memories of the last event. We believe that this first report of the widespread presence of PrLPs in the plant kingdom will pave the way for more detailed and species-specific studies, including experimental validation and characterization of these PrLP candidates as true prions in one, more, or all species investigated in our meta-analysis. In the long run, this knowledge of candidates with beneficial potential prionogenic behavior may help in the generation of stress-resilient rice varieties.

## Data Availability Statement

The original contributions presented in the study are included in the article/Supplementary Material, further inquiries can be directed to the corresponding authors.

## Author Contributions

SS and GY: conceptualization. CK, SG, and GY: visualization and writing – original draft. C and SG: investigation. SS and SS-P: writing, review, and editing. GY, CK, and SS: funding. CK and GY: supervision. All authors have read and approved the manuscript.

## Conflict of Interest

The authors declare that the research was conducted in the absence of any commercial or financial relationships that could be construed as a potential conflict of interest.

## Publisher’s Note

All claims expressed in this article are solely those of the authors and do not necessarily represent those of their affiliated organizations, or those of the publisher, the editors and the reviewers. Any product that may be evaluated in this article, or claim that may be made by its manufacturer, is not guaranteed or endorsed by the publisher.

## Footnotes

1 https://phytozome.jgi.doe.gov/

2 http://congenie.org/

3 http://rice.plantbiology.msu.edu/

4 http://plaac.wi.mit.edu/

5 https://phytozome.jgi.doe.gov/pz/portal.html

6 http://bioinfo.sibs.ac.cn/plant-regulomics/

7 http://revigo.irb.hr/

8 http://bis.zju.edu.cn/ricenetdb/

9 https://genevestigator.com/

10 https://www.ncbi.nlm.nih.gov/geo/geo2r/

11 http://plantregmap.cbi.pku.edu.cn/

## Supplementary Material

Supplementary Figure 1 Gene ontology (GO) enrichment analysis of prion-like proteins (PrLPs) from the organisms used in the study. Heat maps depicting **(A)** molecular functions, **(B)** biological processes, and **(C)** cellular components, enriched in all 39 species. Heatmaps were generated using the R package.

Supplementary Figure 2 Gene ontology (GO) enrichment analysis of rice PrLPs. The *y*-axis represents different GO entries belonging to three categories viz. biological processes (BP), cellular components (CC), and molecular functions (MF). The *x*-axis represents the values of *p* (log10 scale) of the unique GO entries. The circle size indicates the frequency of the terms. The more general terms have a larger circle area. “Plant Regulomics” (http://bioinfo.sibs.ac.cn/plant-regulomics/) and REVIGO (http://revigo.irb.hr/) were used for GO enrichment.

Supplementary Figure 3 Plant prionomes involved in stress memory responses. Expression profile of PrLPs in memory responses pertaining to priming as in **(A)** Arabidopsis Col-0 and freezing-tolerant N-14 genotype (Zuther et al., 2019), under primed conditions (4°C for 3 days followed by 7 days of lag phase at 20°C) and subsequent freezing stress (4°C for 3 days), and in **(B)** *Malus domestica* (GSE123072), upon treatment with BTH (a salicylic acid analog) at 14 days from propagation and subsequently with flg22 (17 days from propagation). PrLP expression profile in memory responses pertaining to recovery phase as in **(C)** *Populus* spp. (Georgii et al., 2019), in response to periodic (three intermittent cycles of 6 days at 33°C and successively increasing drought from 50 to 70%; 2-day recovery in between each cycle) or chronic (33°C and gradually increasing drought at 70% for 22 days) phase of combined drought-heat stress followed by recovery phase (1 week), **(D)** in *Chlamydomonas* (Hemme et al., 2014), when subjected to heat stress (42°C for 24 h) and allowed to recover (25°C for 8 h), and in **(E)** *Arabidopsis* (GSE112161), transcriptome of 4-day-old seedlings when subjected to heat stress (1 h/37°C; 1.5 h/23°C; 45 min/44°C) and then allowed to acclimatize at different time points (4 and 52 h). In general, “primed” refers to exposure to a short span of stress to acclimatize the plant whereas “triggered” means subsequent exposure to stress. Data pertaining to GSE123072 and GSE′61 experiments were analyzed by the GEO2R tool in NCBI (https://www.ncbi.nlm.nih.gov/geo/geo2r/). For panels **(A,C,D)**, the relative expression values were directly taken from the published literature (as described in Supplementary Table 1). The values of log2FC were compared for the respective treatments against the untreated control in each case. The data were plotted as heatmaps using R software.

Supplementary Table 1 Details of experiments used to extract expression profiles of prion-like proteins (PrLPs).

Supplementary Table 2 Stress & memory acclimation genes in plants and their homologs in rice.

Supplementary Table 3 Classification of 39 prion-like candidates into 10 different functional categories.

Supplementary Table 4 Gene ontology (GO) enrichment analysis for all the organisms under study.

Supplementary Table 5 PrLP candidates predicted in the rice prionome with their sub-cellular localizations.

Supplementary Table 6 The 66 rice PrLPs with diurnal expression profiles.

Supplementary Table 7 The rice prionome significant co-expression data sets.

Supplementary Table 8 Rice prionome master gene regulatory network.
