# Supplementary material for: Complex Networks of Prion-Like Proteins Reveal Cross Talk Between Stress and Memory Pathways in Plants
Source: Front Plant Sci. 2021 Jul 26;12:707286. doi: 10.3389/fpls.2021.707286 (PMC8350573; doi:10.3389/fpls.2021.707286)
Supplement: Supplementary Figure 1 — Gene ontology (GO) enrichment analysis of prion-like proteins (PrLPs) from the organisms used in the study. Heat maps depicting (A) molecular functions, (B) biological processes, and (C) cellular components, enriched in all 39 species. Heatmaps were generated using the R package. [file Presentation_1.PPTX]

## Slide 1
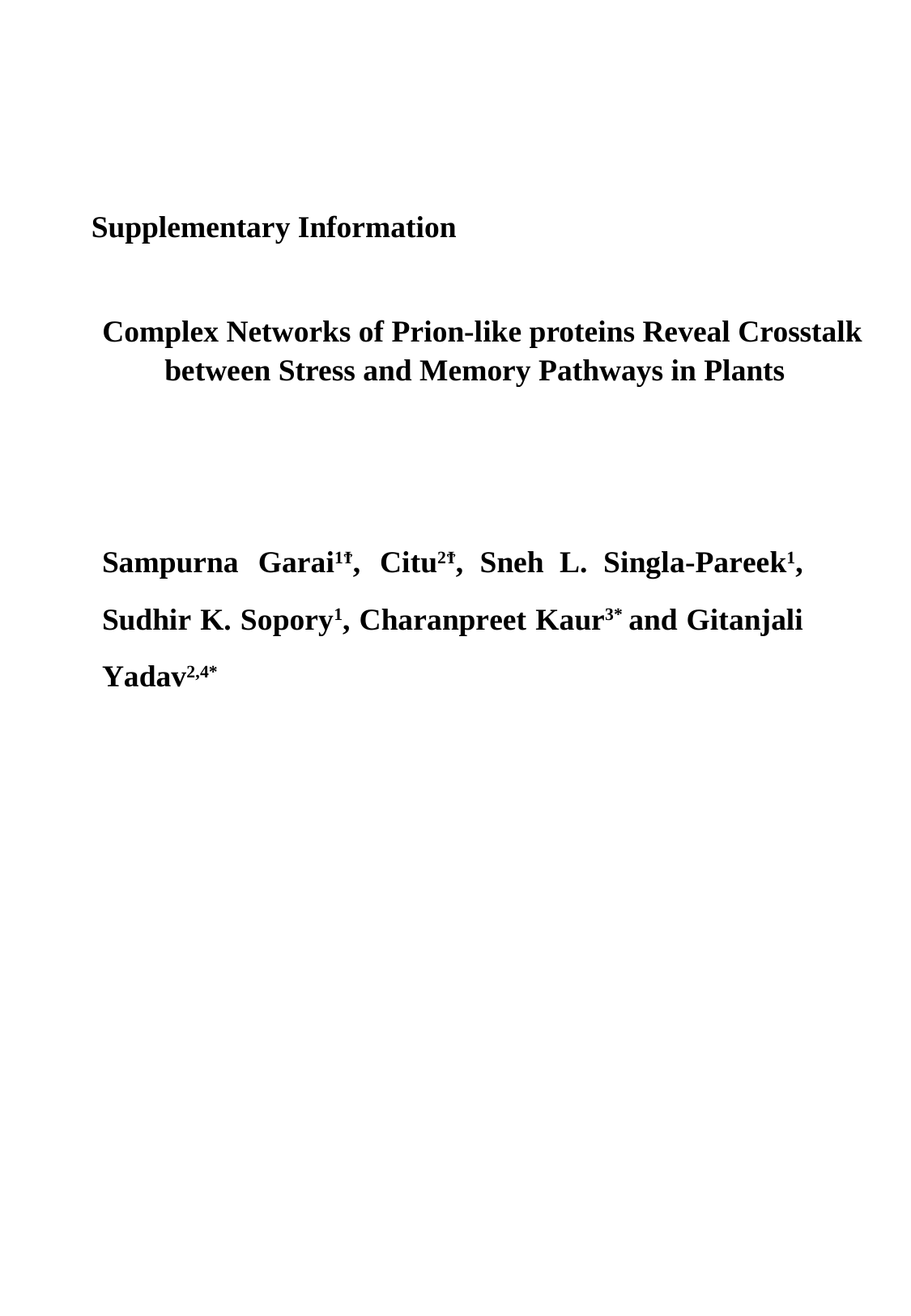

Supplementary Information
 Complex Networks of Prion-like proteins Reveal Crosstalk between Stress and Memory Pathways in Plants
Sampurna Garai1Ϯ, Citu2Ϯ, Sneh L. Singla-Pareek1, Sudhir K. Sopory1, Charanpreet Kaur3* and Gitanjali Yadav2,4*

## Slide 2
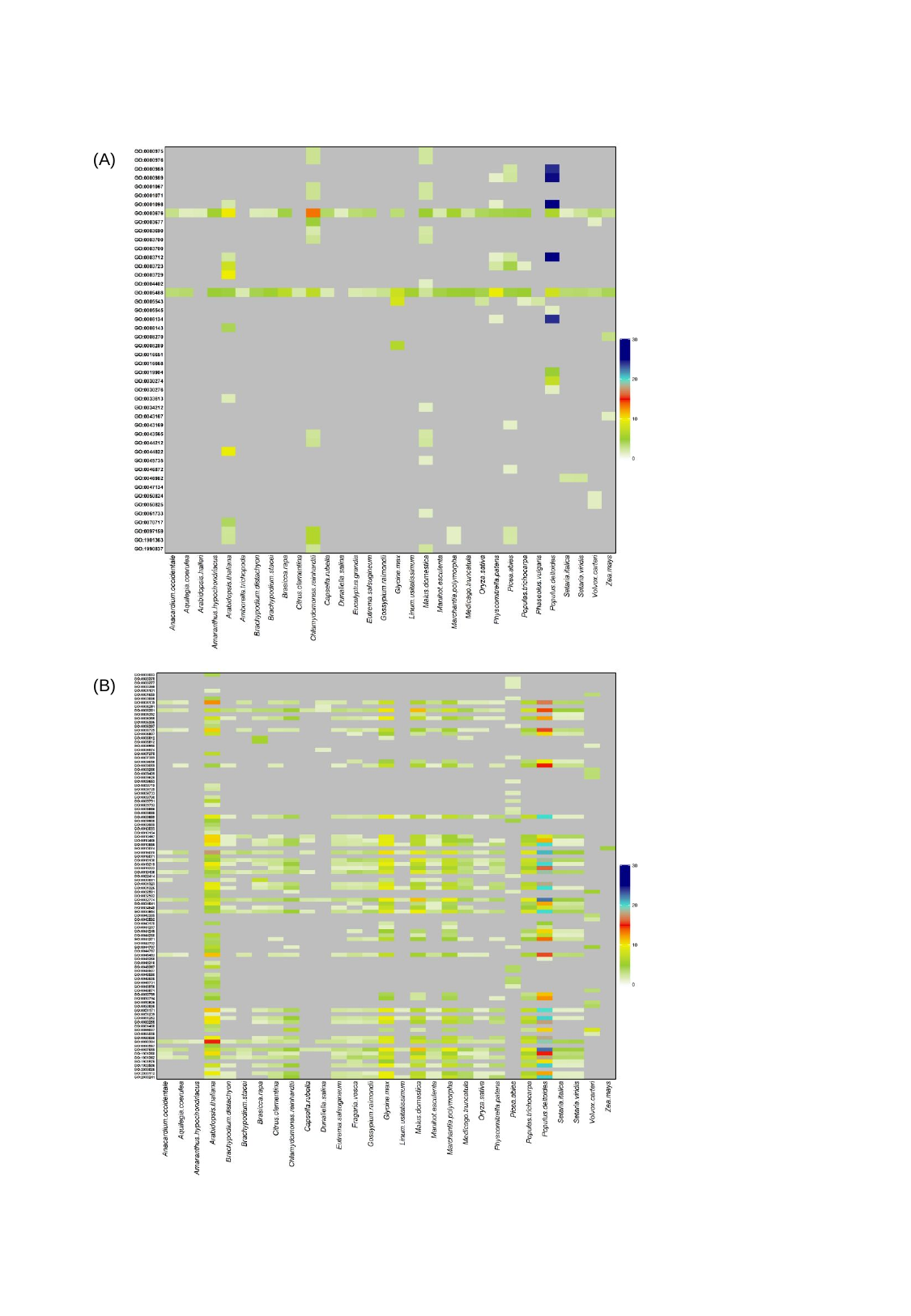

(A)
(B)

## Slide 3
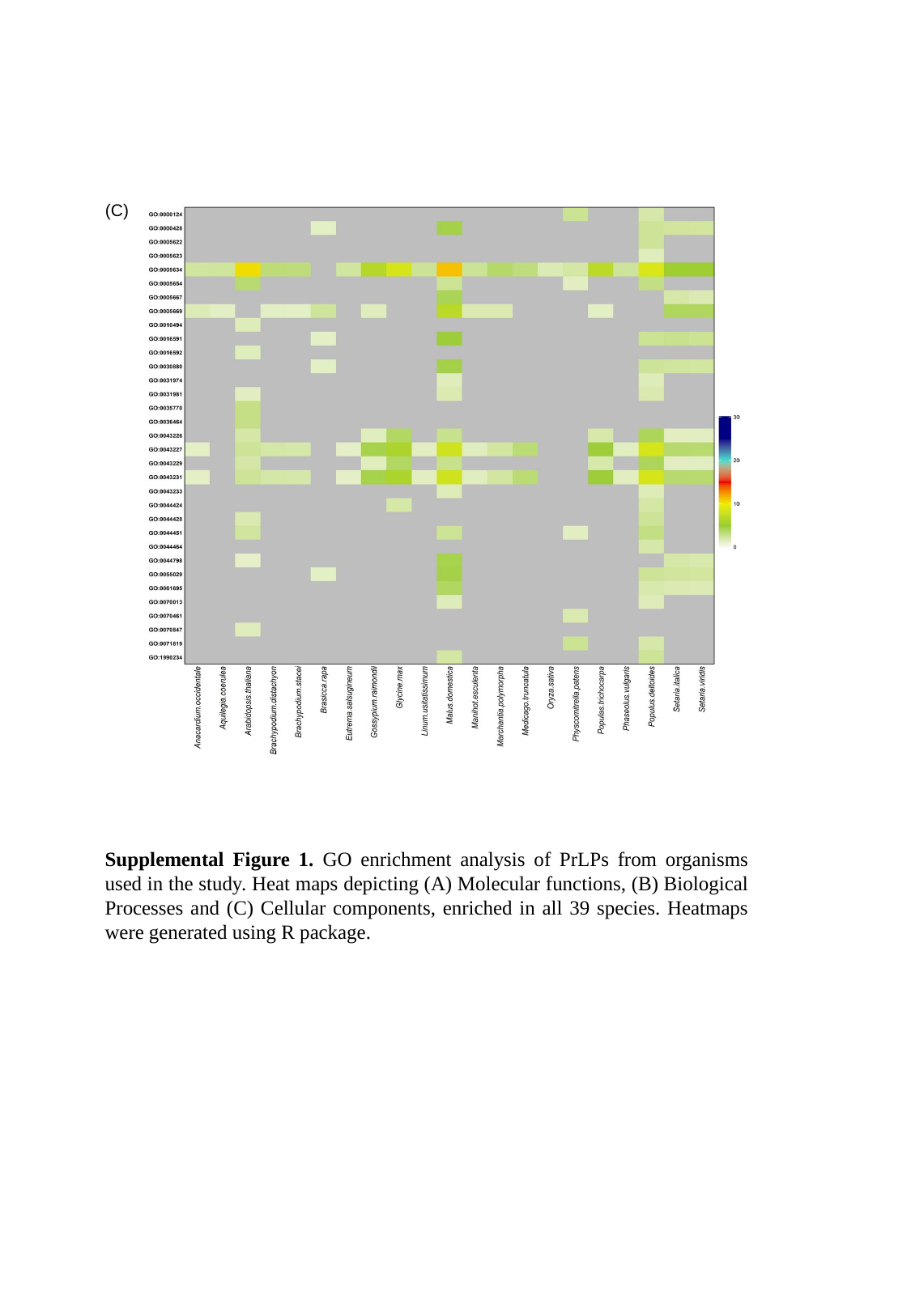

(C)
Supplemental Figure 1. GO enrichment analysis of PrLPs from organisms used in the study. Heat maps depicting (A) Molecular functions, (B) Biological Processes and (C) Cellular components, enriched in all 39 species. Heatmaps were generated using R package.

## Slide 4
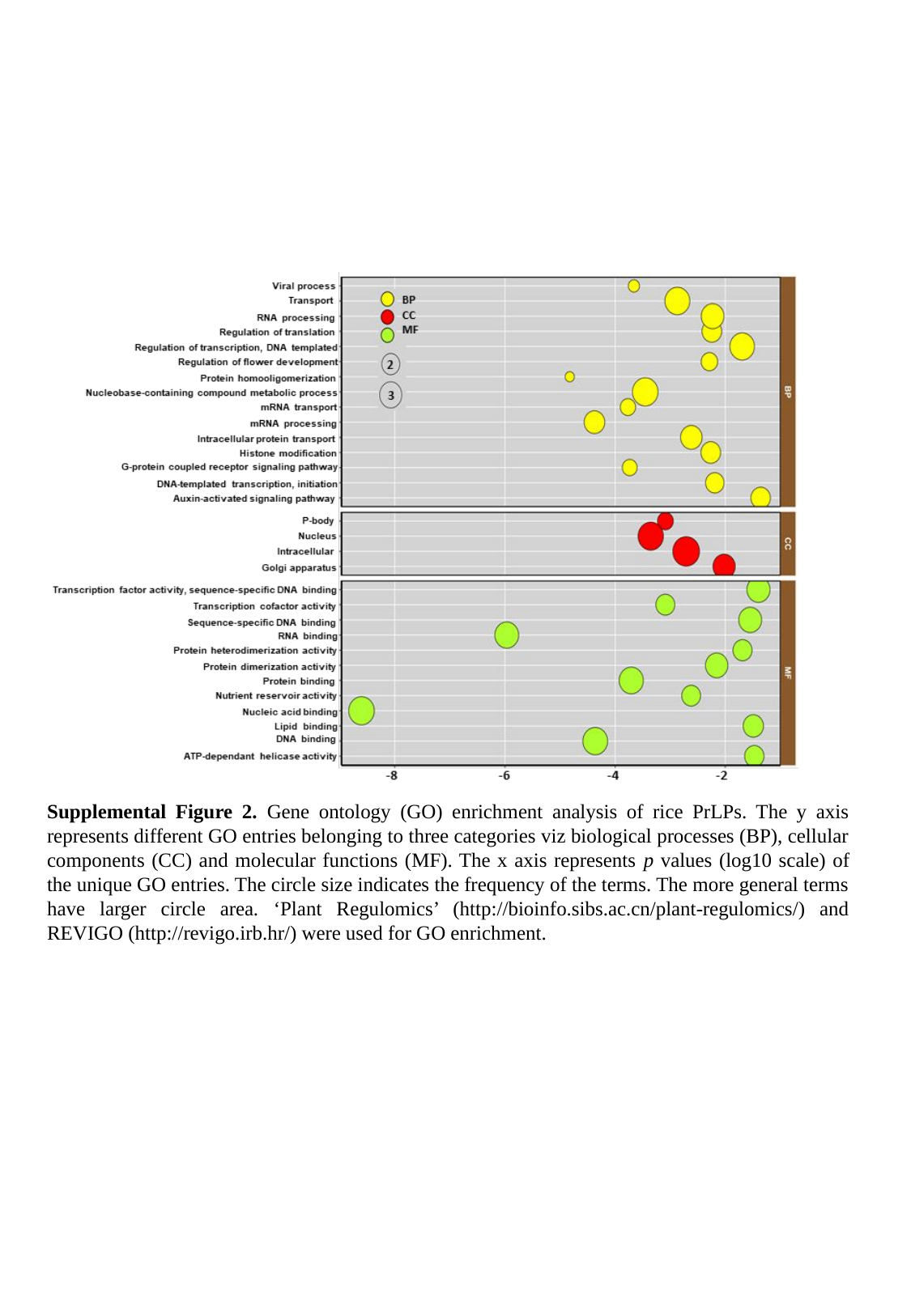

Supplemental Figure 2. Gene ontology (GO) enrichment analysis of rice PrLPs. The y axis represents different GO entries belonging to three categories viz biological processes (BP), cellular components (CC) and molecular functions (MF). The x axis represents p values (log10 scale) of the unique GO entries. The circle size indicates the frequency of the terms. The more general terms have larger circle area. ‘Plant Regulomics’ (http://bioinfo.sibs.ac.cn/plant-regulomics/) and REVIGO (http://revigo.irb.hr/) were used for GO enrichment.

## Slide 5
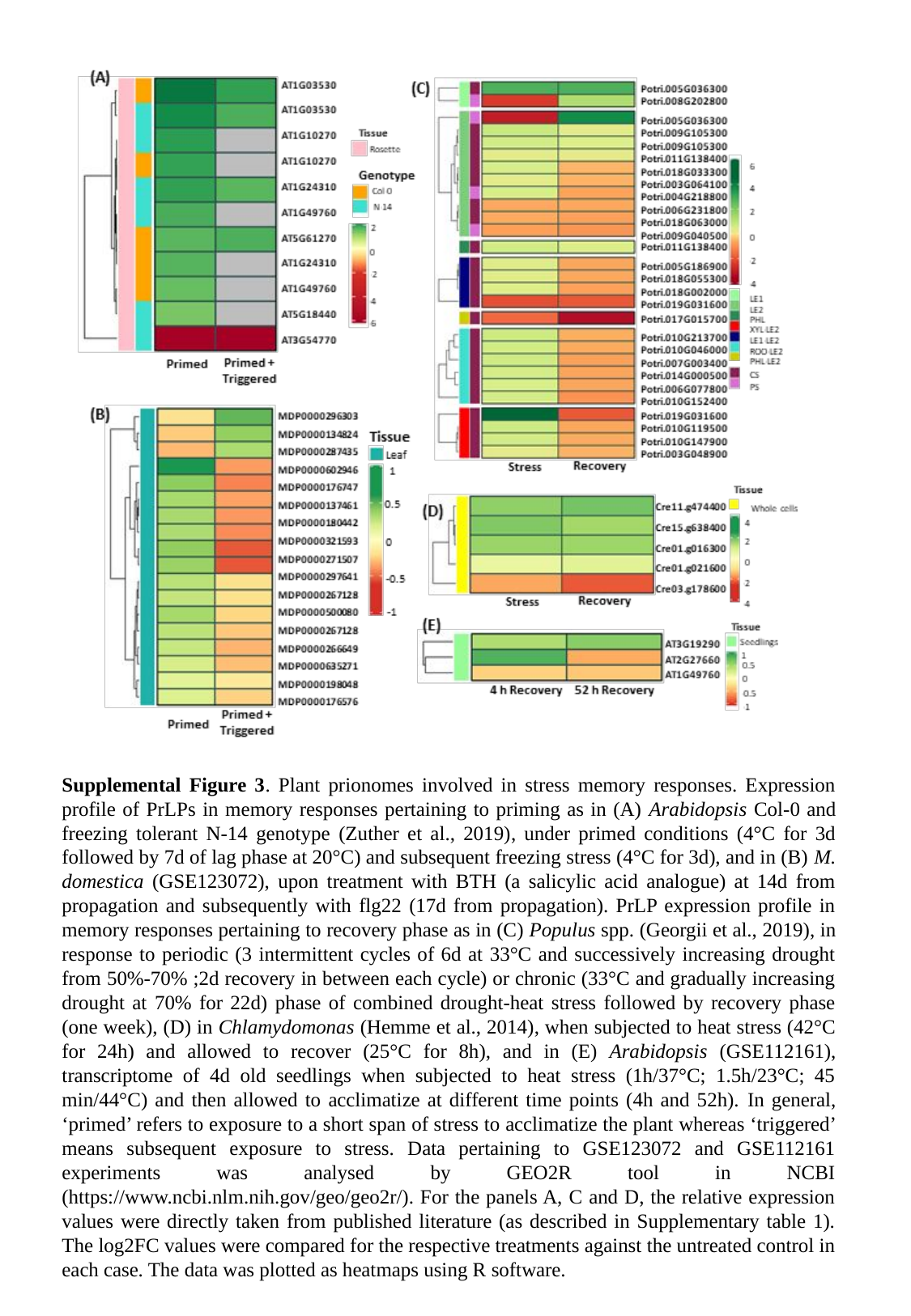

Supplemental Figure 3. Plant prionomes involved in stress memory responses. Expression profile of PrLPs in memory responses pertaining to priming as in (A) Arabidopsis Col-0 and freezing tolerant N-14 genotype (Zuther et al., 2019), under primed conditions (4°C for 3d followed by 7d of lag phase at 20°C) and subsequent freezing stress (4°C for 3d), and in (B) M. domestica (GSE123072), upon treatment with BTH (a salicylic acid analogue) at 14d from propagation and subsequently with flg22 (17d from propagation). PrLP expression profile in memory responses pertaining to recovery phase as in (C) Populus spp. (Georgii et al., 2019), in response to periodic (3 intermittent cycles of 6d at 33°C and successively increasing drought from 50%-70% ;2d recovery in between each cycle) or chronic (33°C and gradually increasing drought at 70% for 22d) phase of combined drought-heat stress followed by recovery phase (one week), (D) in Chlamydomonas (Hemme et al., 2014), when subjected to heat stress (42°C for 24h) and allowed to recover (25°C for 8h), and in (E) Arabidopsis (GSE112161), transcriptome of 4d old seedlings when subjected to heat stress (1h/37°C; 1.5h/23°C; 45 min/44°C) and then allowed to acclimatize at different time points (4h and 52h). In general, ‘primed’ refers to exposure to a short span of stress to acclimatize the plant whereas ‘triggered’ means subsequent exposure to stress. Data pertaining to GSE123072 and GSE112161 experiments was analysed by GEO2R tool in NCBI (https://www.ncbi.nlm.nih.gov/geo/geo2r/). For the panels A, C and D, the relative expression values were directly taken from published literature (as described in Supplementary table 1). The log2FC values were compared for the respective treatments against the untreated control in each case. The data was plotted as heatmaps using R software.
