# Supplementary material for: Complex Networks of Prion-Like Proteins Reveal Cross Talk Between Stress and Memory Pathways in Plants
Source: Front Plant Sci. 2021 Jul 26;12:707286. doi: 10.3389/fpls.2021.707286 (PMC8350573; doi:10.3389/fpls.2021.707286)
Supplement: Supplementary Table 1 — Details of experiments used to extract expression profiles of prion-like proteins (PrLPs). [file Table_1.DOCX]

**Supplementary Table 1. Details of experiments used to extract expression profiles of PrLPs.**

| Details of experiments used to extract ‘stress memory’ data for Supplementary Figure 3 from various spp | | | |
| --- | --- | --- | --- |
| Stress | Description | *Organism* | Reference |
| Cold | Two Arabidopsis accessions were used. The plants that were only cold primed were showing lesser freezing tolerance than the plants that were exposed to cold priming at 4°C, a lag phase at 20°C, and a second triggering cold stress (4°C). | *Arabidopsis thaliana*  (Col-0 and freezing tolerant genotype N14) | Zuther et al., 2019  Data retrieved from Supplementary  table: 6 |
| Salicylic Acid | The microarray based transcription profiles were generated to identify the primed genes resulting after the treatment with SA analogue BTH at 14d from propagation and subsequently with flg22 at 17 d from propagation in two biological replicates. | *Malus domestica* | GSE123072  GEO2R used to extract log2FC values |
| Drought-Heat | Periodic (3 intermittent cycles of 6d at 33°C and successively increasing drought from 50%-70%) versus chronic (33°C and gradually incresing drought at 70% for 22d) drought-heat stress conditions were compared in gray poplar. The phenotypic and transcriptomic effects were measured during stress and after recovery. | *Populus spp*  Gray poplar (Populus× canescens) | Georgii et al.,2019  Data retrieved from Supplementary: data set 1 |
| Heat | The cells were shifted from 25 to 42°C for upto 24 h and then back to 25°C for upto24 h for recovery (samples were harvested at different time points) to study the acclimation process to long-term heat stress. | *Chlamydomonas reinhardtii* | Hemme et al., 2014  Data retrieved from Supplementary table: DS2 |
| Heat | Microarray transcriptome profiling of 4d old seedlings when subjected to heat stress (1h/37°C;1.5h/23°C;45 min/ 44°C and then allowed to acclimatize at different time points (4 h after acclimation 52 h after acclimation, and 4 h non-treated control) was done. | *Arabidopsis thaliana* | GSE112161  GEO2R used to extract log2FC values |
| Details of experiments used to extract data for ‘stress’ expression profiles of rice PrLPs from Genevestigator for Figure 5 (abiotic and biotic treatments). | | | |
| Biotic stress | Plants were incubated vertically at 30°C in the dark for 4 d to promote root growth and then vertically with a 16-h-light/8-h-dark photoperiod at 28/24°C for 6 d. M. oryzae mycelium was cultured at 26°C on solid complete medium with a 16-h-light/8-h-dark photoperiod, and conidia were harvested from 8-d-old cultures by rinsing mycelium several times. Infection assays were conducted on root. | Host: Oryza sativa  Pathogen: *Magnaporthe oryzae* | GSE18361  log2FC values retrieved |
| Biotic stress | Xoo was used to infect 35-day old plants (cv. IR24 and IRBB21) and the leaf samples were collected at 24 h and 96 h after infection. | Host: Oryza sativa  Pathogen: Xanthomonas oryzae pv. Oryzae (Xoo) :strain PXO71) | GSE43050  log2FC values retrieved |
| Cold,  Salinity, Drought | The seedlings were transferred to a beaker containing 200 mM NaCl solution for 3 h. For desiccation, rice seedlings were dried for 3 h between folds of tissue paper at 28±1 degree C. For cold treatment, the seedlings were kept at 4±1 degree C for 3 h. | *Oryza sativa* | GSE6901  log2FC values retrieved |
| Drought | Hydroponically grown seven-day-old seedlings were subjected to drought stress by placing them on 3 mm Whatmann sheets under light for 3 h and 6 h at 28±1oC. Samples were analyzed after 3 h and 6 h of dehydration stress. | *Oryza sativa* | GSE41647  log2FC values retrieved |
| Heat- Salinity- Drought- Cold | The seedlings were grown under the condition of 14 h light/10 h dark at 28–30 °C and then transferred to 42 °C under light for heat treatment. Two weeks old seedlings were cultured in water supplied with 200 mM NaCl for salt treatment. For drought treatment, the roots were dried and placed under normal growth conditions. Seedlings were kept at 4 °C without light for cold treatment. The shoots were harvested at 0, 1, 3, 10 h after each treatment. | *Oryza sativa* | GSE14275  log2FC values retrieved |
| Salinity | The seedlings were grown at NaCl (salt) concentration of 140 mM and salt+ABA (140 mM NaCl + 10 μM ABA). The seedlings were grown at 29/22 °C day/night temperatures and a minimum relative humidity of 70%. | *Oryza sativa* | GSE58603  log2FC values retrieved |
| Development | 39 tissues/organs covering the life cycle of the rice from two indica varieties Minghui 63 and Zhenshan 97 were collected and the Affymetrix GeneChip Rice Genome Array was used to investigate the transcriptomes of these organs. | *Oryza sativa* | GSE19024  log2FC values retrieved |
| Low temperature stress | Three week old plants were subjected to 4°C for varying periods | Oryza sativa, ssp. japonica, cv. Jumli Marshi (JM) and ssp. indica, cv. IR64 (IR64) | E-MEXP-3718 |
| Drought | Water was withheld to 14 d old seedlings till leaf rolling was observed | *Oryza sativa:* IR64 and N-22 | E-MEXP-2401 |
